# Establishment of gender- and age-specific reference intervals for serum liver function tests among the elderly population in northeast China: a retrospective study

**DOI:** 10.11613/BM.2022.020707

**Published:** 2022-06-15

**Authors:** Zeyu Sun, Jiatong Chai, Qi Zhou, Jiancheng Xu

**Affiliations:** 1 Department of Laboratory Medicine, First Hospital of Jilin University, Changchun, China; 2 Department of Pediatrics, First Hospital of Jilin University, Changchun, China

**Keywords:** gender, age, liver function, elderly population, reference interval

## Abstract

**Introduction:**

Reference intervals (RIs) for younger population may not apply to the elderly population. The aim of this study was to establish gender- and age-specific RIs for serum liver function tests among the elderly population and to compare with younger population RIs currently used in China and other countries.

**Materials and methods:**

This was a retrospective study, and subjects (≥ 18 year-old) were recruited from the laboratory information system (LIS) at the First Hospital of Jilin University between April 2020 and April 2021. The following parameters were collected: aspartate aminotransferase (AST), alanine aminotransferase (ALT), gamma-glutamyltransferase (GGT), alkaline phosphatase (ALP), total protein (TP), albumin (ALB), total bilirubin (TBIL), and direct bilirubin (DBIL). The Tukey method was used to eliminate outliers. Reference intervals were established by the nonparametric method.

**Results:**

A total of 23,597 healthy individuals were enrolled in the study. From all parameters AST, ALT, TP and ALB required no gender partition, while ALT, GGT, TP, ALB and DBIL required different partitions for age. Activities and concentrations of ALT, ALB, and TP showed a downward trend in the elderly aged 60-89. In contrast, DBIL showed a gradual upward trend.

**Conclusion:**

The RIs for liver function tests among healthy elderly population were different from those among young population in China. There were apparent gender and age differences in the RIs of liver function for elderly and significant differences compared with national standards and RIs in other countries. Therefore, it is necessary to establish gender- and age-specific RIs for serum liver function tests among the elderly population.

## Introduction

Biological reference intervals (RIs) are an important basis for clinicians to interpret patients’ test results. The direct method for establishing RIs has been recommended as the gold standard in recent years (*1*). However, a new indirect method based on data mining techniques is gaining attention because it saves time and money and guarantees a sample size for each interval when stratifying (*2*, *3*). Inappropriate RIs used by laboratories could potentially lead to misdiagnoses, inaccurate therapeutic approaches and duplicate detection. Reference intervals are influenced not only by personal factors such as gender, age, and living habits but also by ecological factors such as altitude, climate, and ethnicity (*4*). Hence, each laboratory should establish its own RIs according to defined procedures.

According to guideline C28-A3c issued by the Clinical Laboratory Standard Institute (CLSI), established RIs should reflect subgroup distinctions, such as gender and age (*5*). Subgroup differentiation is necessary for particular groups, such as children, younger populations and elderly populations. According to the 7th nationwide census of the Chinese population in 2020, China had 264 million people aged ≥ 60 years, accounting for 18.7% of the total population (*6*). With an increasing aged population in China, the demands for medical services are increasing, and the aged population seeks medical security more often than younger individuals. Compared with younger individuals, elderly individuals not only have poor physical function and nutritional status, but also a higher risk of chronic diseases such as hypertension and diabetes (*7*). At present, most countries or regions do not systematically publish RIs for elderly individuals. Most clinicians or laboratories still use adult RIs. Many studies have shown that adult RIs are not suitable for older people’s health assessments (*8*). For example, the aging process is closely associated with a lot of degenerative changes in the liver, in which liver structure and cell function are observed to decline (*9*). In addition, aging is a major risk factor for the development and prognosis of several chronic liver diseases and conditions. The decreased ability of older livers to regenerate and to tolerate transplantation leads to increased risk of death from chronic liver disease (*10*). Physiological or pathological changes make it less feasible for older adults to share the same RIs with younger adults. Furthermore, some routine clinical parameters show wider RIs for the elderly. For example, wider RIs have generally been reported for sodium, potassium, calcium and C-reactive protein (CRP) in elderly individuals (*11*). In addition, few RIs for haematological and biochemical indices can be applied directly to elderly subjects without finding excess out-of-range values (*8*).

Biochemical tests of liver function are routine tests for the assessment of liver status (*12*). Important parameters, such as aspartate aminotransferase (AST), alanine aminotransferase (ALT), gamma-glutamyltransferase (GGT), alkaline phosphatase (ALP), total protein (TP), albumin (ALB), total bilirubin (TBIL), and direct bilirubin (DBIL), play a suggestive role in the early stage of hepatobiliary diseases. Furthermore, recent studies have shown that AST and ALT are closely related to the metabolism of cancer cells and the prognosis of different types of cancer (*13*). In addition, GGT and ALP are independently related to renal prognosis (*14*). It is of great significance to establish accurate and reliable RIs for these parameters. There were many studies on RIs that included these parameters in China (*2*, *15*, *16*). Reference intervals for people older than 60 years of age were also reported, but the exact partition age of RIs was not established.

Therefore, this study aimed to establish gender- and age-specific RIs for serum liver function tests in the elderly population, and to compare with younger population RIs currently used in China and other countries.

## Materials and methods

### Subjects

**Figure 1:**
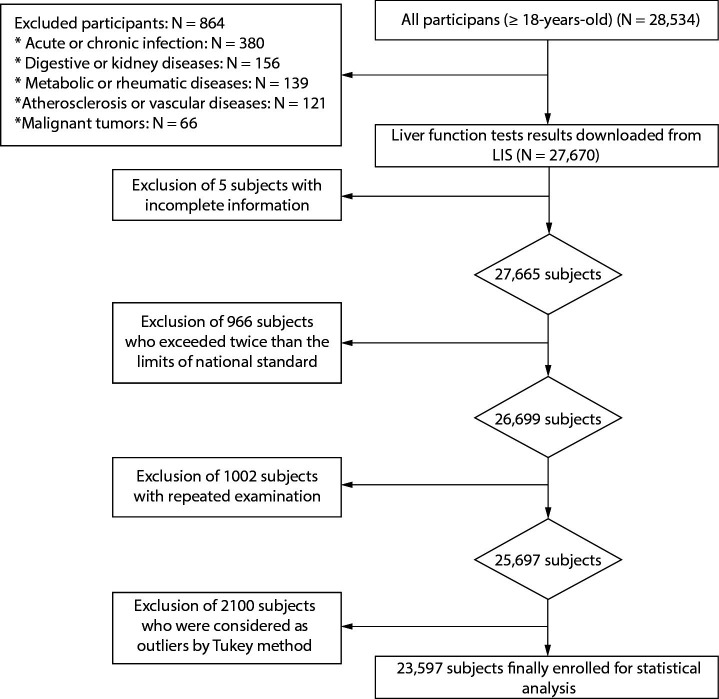
The specific protocol for data removal.

We conducted a retrospective study focusing on liver function in the elderly. All individuals (≥ 18-year-old) who had undergone biochemical testing in the health examination center were enrolled in the study from April 2020 to April 2021. The data were collected from LIS of the First Hospital of Jilin University. To improve the accuracy of the RIs in this study, we first defined apparently healthy individuals according to the protocol provided by the International Federation of Clinical Chemistry and Laboratory Medicine (IFCC) Committee on Reference Intervals and Decision Limits (*17*). The following exclusion criteria were implemented: positive for human immunodeficiency virus antibodies; a recent history of acute or chronic infection; digestive, kidney, metabolic or rheumatic diseases; atherosclerosis or vascular disease; malignant tumors; and burns or muscle damage. The specific protocol for data removal is shown in Figure 1. Finally, 23,597 subjects were enrolled in this study, including 20,048 subjects (18-59 years old) and 3549 subjects (60-89 years old).

This retrospective observational study was approved by the Ethics Committee of the First Hospital of Jilin University (NO: 2021-543). All data was obtained from the LIS, and only medical records were analysed. The need for informed consent (both written and oral) was waived by the Committee. This waiver does not adversely affect the rights and welfare of the subjects. All procedures involving human participants were performed in accordance with the ethical standards of the Ethics Committee for Clinical Research of the First Hospital of Jilin University and with the Declaration of Helsinki and its later amendments.

### Methods

All participants fasted for 12 hours overnight and avoided alcohol in-take during the 24 hours before blood collection. Blood samples (4 mL) were collected into BD Vacutainer Serum Separator II Advance Tubes (SST II) (Becton, Dickinson and Company, Franklin Lakes, USA). After storage at room temperature (approximately 22 °C) for 30 minutes, the samples were centrifuged at 3000 r/min for 10 minutes and then tested within 2 hours according to the manufacturer’s instructions.

All biochemical parameters were analysed by Hitachi 7600-210 automatic analyser (Hitachi High-Technologies, Tokyo, Japan). The measured parameters and methods were as follows: AST (UV-MDH method), ALT (UV-LDH method), GGT (l-γ-glutamyl-3-carboxy-4-nitranilide method), ALP (AMP buffer method), TP (biuret reaction), ALB (bromocresol green method), TBIL and DBIL (DCA method). All samples were tested within normal quality control.

All data were stored in the LIS. The system is responsible for daily data processing, including sample collection, sample data reception, data processing, report reviews, report releases, and report queries. It is also used for patient information browsing, historical data comparisons, historical data querying, *etc*.

### Statistical analysis

The Kolmogorov-Smirnov test was performed to assess the distribution of data. If the data showed a non-Gaussian distribution, the Box-Cox transformation method was used to transform the data to an approximately Gaussian distribution. The Tukey method was used to eliminate outliers. After calculating the values of the 25^th^ and 75^th^ percentiles of the data distribution (P_25_, P_75_) and IQR (interquartile range), the lower limits (LL) and upper limits (UL) were calculated as P_25_ − 1.5 x IQR and P_75_ + 1.5 x IQR. Any data outside of this range were regarded as outliers. The Harris and Boyd method was used to determine whether it was necessary to partition the reference values for gender. Z > Z* was considered significant. The difference between the younger and elderly population, different age subgroups in the elderly population were compared using the Mann-Whitney U-test. P-values < 0.05 were considered statistically significant. The lambda-mu-sigma (LMS) method was used to analyse the dynamic changes in analytes. The LL and UL of RIs (2.5^th^ and 97.5^th^ percentiles) were calculated by the nonparametric method. The relative deviations of the LL and UL of each analyte’s RIs with the national standard and other studies were calculated for comparison with the corresponding reference change value (RCV) (). The analysis coefficient of variation was CVa, and CVi was the intraindividual biological variation coefficient. They were obtained through the European Federation of Clinical Chemistry and Laboratory Medicine (EFLM) Biological Variation database and Westgard website (*18*, *19*). Z was the probability of difference. If the relative deviation exceeded the RCV, the difference between them was statistically significant. Data were analysed using Statistical Package for the Social Sciences (SPSS) version 23.0 (IBM, Armonk, USA), Minitab 19 software (State College, USA), GraphPad Prism 8 (GraphPad Prism Software Inc, San Diego, USA), and LMS ChartMaker Light software (Medical Research Council, Cambridge, England).

## Results

### Distribution and elimination of enrolled data

Except for TP and ALB, the other analytes showed skewed distributions, and were transformed into approximately Guassian distributions by the Box-Cox transformation method. In addition, 2100 data points were eliminated by the Tukey method. Finally, 23,597 subjects met the inclusion criteria, and the ratio of male to female was 1:1.18. Each subgroup comprised as the following: men < 60 years old (N = 8996 and median age = 43 (18-59) years); men ≥ 60 years old (N = 1820 and median age = 65 (60-89) years); women < 60 years old (N = 11,052 and median age = 38 (18-59) years); and women ≥ 60 years old (N = 1729 and median age = 65 (60-89) years).

### Comparison of analytes’ concentrations between the young and the elderly

The median and RIs for each analyte in the < 60 and ≥ 60-year-old groups divided by gender are summarized in Table 1. Except for AST and ALP, a significant difference in other analytes was observed between the younger and elderly populations for males (P < 0.001). Furthermore, all analytes were significantly different between the younger and elderly populations for females (P < 0.001).

**Table 1:** Gender- and age-specific medians and RIs for 8 liver function parameters of healthy adults

	**Age group** **(years)**	**N**	**Median (IQR)**	**RIs**	**Lower 90% CI**	**Higher 90% CI**	**P**
**Males**							
AST (U/L)	18-59	8996	20.5 (17.6-24.6)	13.4-38.4	13.3-13.6	37.7-39.2	0.392
	≥ 60	1820	20.4 (17.7-24.0)	13.5-38.7	13.3-13.6	36.7-41.1	
ALT (U/L)	18-59	8996	22.9 (16.9-32.3)	10.0-65.6	9.8-10.2	64.2-66.6	< 0.001
	≥ 60	1820	18.2 (14.1-23.9)	8.8-46.7	8.5-9.4	44.2-49.1	
GGT (U/L)	18-59	8996	27.1 (18.8-41.7)	10.3-94.4	10.0-10.5	92.4-96.4	< 0.001
	≥ 60	1820	22.5 (16.3-32.7)	9.1-81.5	8.7-9.7	75.9-86.1	
ALP (U/L)	18-59	8996	60.1 (51.6-70.8)	37.5-95.9	37.2-37.9	95.0-96.9	0.113
	≥ 60	1820	60.5 (51.8-72.0)	38.3-99.3	36.5-39.3	97.3-102.5	
TP (g/L)	18-59	8996	75.0 (72.5-77.3)	67.9-81.0	67.8-68.2	80.9-81.1	< 0.001
	≥ 60	1820	74.1 (71.4-76.7)	66.8-82.2	66.5-67.2	81.8-82.6	
ALB (g/L)	18-59	8996	45.0 (43.4-46.4)	40.5-49.3	40.4-40.5	49.2-49.4	< 0.001
	≥ 60	1820	43.2 (41.7-44.7)	38.6-47.6	38.4-38.8	47.4-47.8	
TBIL (μmol/L)	18-59	8996	16.3 (13.1-20.6)	8.4-32.1	8.2-8.5	31.5-32.6	< 0.001
	≥ 60	1820	17.3 (14.0-21.7)	9.0-35.7	8.5-9.4	34.4-36.7	
DBIL (μmol/L)	18-59	8996	3.2 (2.5-4.1)	1.5-6.6	1.4-1.5	6.5-6.6	< 0.001
	≥ 60	1820	3.6 (2.8-4.6)	1.6-7.9	1.5-1.7	7.6-8.3	
**Females**							
AST (U/L)	18-59	11,052	17.2 (15.1-20.3)	12.1-32.9	12.1-12.2	32.1-33.5	< 0.001
	≥ 60	1729	20.4 (17.7-23.9)	13.7-41.5	13.2-14.0	39.1-44.1	
ALT (U/L)	18-59	11,052	13.4 (10.3-18.4)	6.7-42.9	6.6-6.8	41.3-44.4	< 0.001
	≥ 60	1729	16.0 (12.6-21.6)	8.1-47.2	7.7-8.4	44.5-50.2	
GGT (U/L)	18-59	11,052	13.2 (10.1-18.8)	5.9-49.9	5.8-6.0	48.1-51.0	< 0.001
	≥ 60	1729	16.4 (12.4-24.2)	7.2-61.6	6.7-7.6	52.9-66.4	
ALP (U/L)	18-59	11,052	52.6 (44.1-64.2)	32.9-95.3	32.6-33.2	94.4-96.4	< 0.001
	≥ 60	1729	69.3 (59.4-81.7)	41.7-115.8	39.9-43.4	112.6-119.6	
TP (g/L)	18-59	11,052	74.6 (72.2-77.0)	67.9-81.0	67.7-68.0	80.9-81.1	< 0.001
	≥ 60	1729	74.7 (72.0-77.4)	67.0-82.5	66.7-67.4	82.1-82.9	
ALB (g/L)	18-59	11,052	43.4 (41.9-44.9)	39.1-48.1	39.0-39.2	47.9-48.2	< 0.001
	≥ 60	1729	42.7 (41.3-44.2)	38.5-47.1	38.3-38.7	46.9-47.2	
TBIL (μmol/L)	18-59	11,052	13.3 (10.8-16.8)	7.2-27.5	7.1-7.3	27.1-28.0	< 0.001
	≥ 60	1729	14.1 (11.7-17.2)	8.0-27.1	7.7-8.2	25.6-28.6	
DBIL (μmol/L)	18-59	11,052	2.5 (1.9-3.3)	1.2-5.7	1.2-1.3	5.6-5.8	< 0.001
	≥ 60	1729	2.3 (1.9-3.0)	2.3-5.2	2.3-2.4	5.1-5.4	
RIs - reference intervals. IQR - interquartile ranges. AST - aspartate aminotransferase. ALT - alanine aminotransferase. GGT - gamma-glutamyltransferase. ALP - alkaline phosphatase. TP - total protein. ALB – albumin. TBIL - total bilirubin. DBIL - direct bilirubin.							

### Gender-specific distributions of liver function tests in the elderly

**Figure 2:**
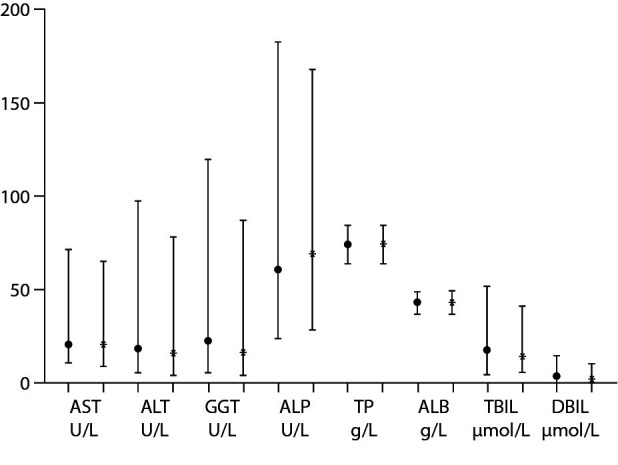
Gender-specific distributions of liver function tests in the elderly. The distance between the top and the bottom horizontal lines represents the range of reference values. The dots represent male and the asterisks represent female, respectively. AST - aspartate aminotransferase. ALT - alanine aminotransferase. GGT - gamma-glutamyltransferase. ALP - alkaline phosphatase. TP - total protein. ALB – albumin. TBIL - total bilirubin. DBIL - direct bilirubin.

The study revealed a strong gender dependency in the RIs of GGT, TBIL, and DBIL. The median, 2.5^th^ and 97.5^th^ percentiles (median (P_2.5_-P_97.5_)) of GGT, TBIL and DBIL in males (22.5 (9.1-81.5) U/L, 17.3 (8.9-35.7) μmol/L, 3.6 (1.6-7.9) μmol/L, respectively) were significantly higher than those in females (16.4 (7.2-61.6) U/L, 14.1 (8.0-27.1) μmol/L, 2.3 (1.1-5.2) μmol/L, respectively; z* = 11.54, z = 13.80, 18.41, 29.17, respectively). Activity of ALP (Z = 15.22) appeared to be the opposite, the values of which were significantly higher in females than in males (69.3 (41.7-115.8) U/L *vs*. 60.5 (38.3-99.3) U/L). There was no significant gender difference in AST, ALT, TP or ALB (Z = 1.01, 5.17, 4.20, 6.38, respectively). Gender-specific distributions of liver function tests in the elderly are shown in Figure 2.

### Age-specific distributions of liver function tests in the elderly

All subjects were stratified according to age, with group intervals of 10 years (males/females of 60-69 years old: N = 1373/1254, 70-79 years old: N = 352/380, 80-89 years old: N = 95/95). There was no significant difference among age subgroups for AST (P = 0.076), GGT in females (P = 0.118), ALP (P = 0.323 in males and P = 0.445 in females) and TBIL (P = 0.197 in males and P = 0.491 in females). Hence, age partitions were not required for these analytes.

**Figure 3:**
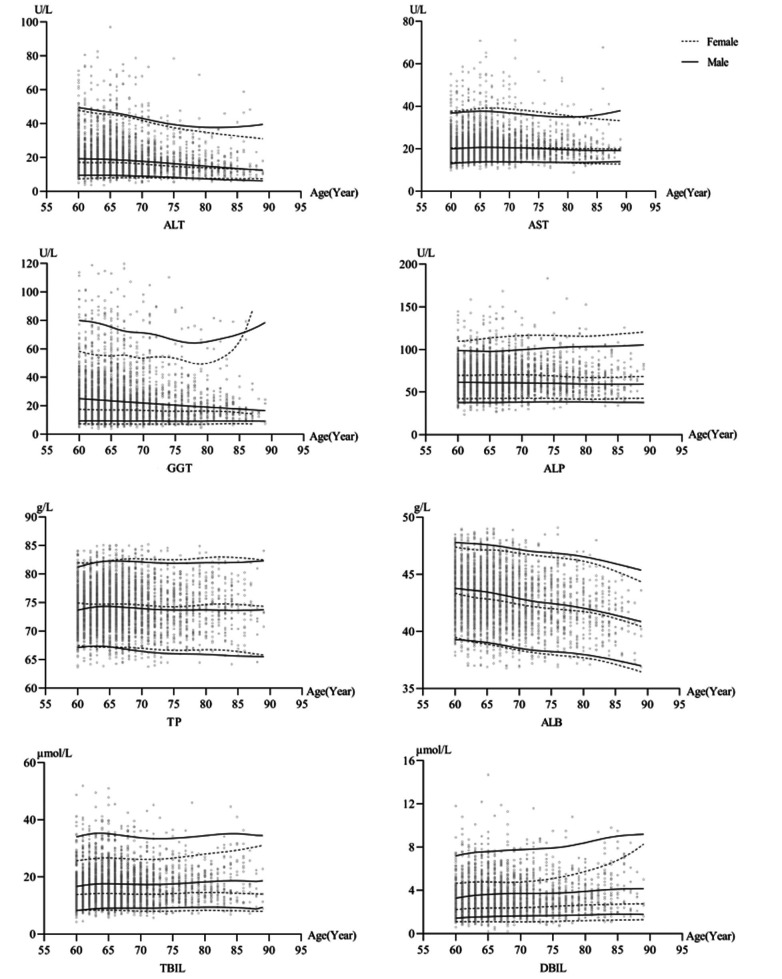
Gender- and age-specific continuous percentile curves of all analytes. The sequences of each pair of curves from top to down are expressed as P_97.5_, P_50_ and P_2.5_ for males and females. AST - aspartate aminotransferase. ALT - alanine aminotransferase. GGT - gamma-glutamyltransferase. ALP - alkaline phosphatase. TP - total protein. ALB – albumin. TBIL - total bilirubin. DBIL - direct bilirubin.

The percentile curves of each analyte in elderly individuals are shown in Figure 3. The trend of all analytes should be interpreted by the 50^th^ percentile curve (P_50_) in the figure because it is the most stable and representative. Activity and concentrations of ALT, ALB, and TP showed a downward trend in the elderly aged 60-89. In contrast, DBIL showed a gradual upward trend. The median of AST, ALP, and TBIL remained relatively stable throughout this age range.

### Establishment of RIs in Northeast China and comparison with currrently used RIs

**Figure 4:**
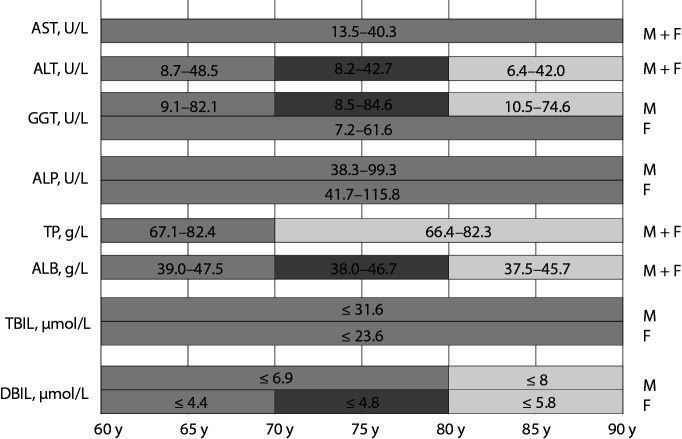
Gender- and age-specific RIs of liver function tests in the healthy elderly population. M – male. F - female. AST - aspartate aminotransferase. ALT - alanine aminotransferase. GGT - gamma-glutamyltransferase. ALP - alkaline phosphatase. TP - total protein. ALB – albumin. TBIL - total bilirubin. DBIL - direct bilirubin. RIs - reference intervals.

The RIs established by the nonparametric method are shown in Figure 4. Reference intervals for most analytes needed to be divided according to gender, age or both. Apparently, AST, ALT, TP and ALB showed no need for gender partition in the whole group. ALP and TBIL required no age partition, whereas GGT and DBIL in males and females displayed a distinct age dependency. Although differences in concentrations across ages were relatively minor, 2 different age partitions were required in males for DBIL. GGT in males and DBIL in females required 3 different age partitions.

The Supplemental table lists the RIs obtained in this study and RIs derived from national standards (*20*-*22*). Studies in Canada, Denmark and Asmara, Eritrea were also used to compare the differences in RIs among regions (*23*-*25*). The relative deviations of the LL and UL of each analyte’s RIs with the national standard and other studies were also included. The relative deviation between RIs of ALT in males aged 70-89, the UL of ALB RIs and RIs of ALP in males and Chinese national standards were higher than the RCV. Relative deviations for other analytes were less than the RCV. The specific comparison between the results of this study and other studies is shown in the Supplemental table.

## Discussion

Our study enriches studies on RIs of liver function tests in Chinese elderly. The establishment of RIs in the elderly is one of the most difficult tasks for laboratories because it is difficult to directly collect blood samples from healthy elderly individuals. However, the indirect method of analysing the existing data in the hospital database with a mathematical statistical model perfectly solves the problem. Therefore, to the best of our knowledge, this is the first study to establish RIs for liver function tests in the elderly based on the LIS by the indirect method in northeast China. Furthermore, the results of this study confirmed that no gender partitions were required for AST, ALT, TP and ALB. Except for AST, GGT in females, ALP and TBIL, the other analytes required age partitions.

The main findings included the facts that RIs for serum TP and ALB didn’t require gender partitioning, which was consistent with national standard, Canada and Denmark (*23*, *24*). The downward tendency in serum TP concentrations may be due to the decrease in the volume and number of liver cells with age. Similarly, abnormal liver function, reduced protein intake and poor absorption are more likely to occur in the elderly, resulting in decreased TP synthesis ability (*26*). Elderly individuals have less physical strength, and a high risk of fracture and are more susceptible to infections, which may increase the consumption of a large number of serum proteins. We found that the relative deviation of the UL or LL of the RIs for ALB was higher than the RCV compared with the national standard, and the Denmark and Asmara studies, the reason may be related to population, ethnicity, *etc.* (*27*).

The data also demonstrated the same results as those of Asmara, namely, that AST and ALT did not reveal a distinct gender dependency in the elderly (*25*). The results were also in agreement with already established knowledge that ALT activities are not affected by gender or metabolic factors (*28*). However, the RIs were slightly different. The relative deviations of the LL of AST and ALT obtained in this study were higher than the RCV compared with the Canada and Denmark studies. We assume that this may be due to differences in laboratory equipment, detection systems, *etc*. (*4*).

Gender and age partitions were required for the remaining four analytes. GGT in males was higher than that in females, which was consistent with other studies (*20*, *23*). One study has shown that high activities of GGT are found in kidney, prostate, pancreas, and hepatobiliary tissues, and higher activity in males maybe due to high enzyme concentrations in prostate tissue (*29*). Furthermore, GGT is also a marker of alcohol intake. Although we did not exclude factors related to alcohol consumption in the enrolled participants, several studies have shown that GGT is closely related to fatty liver, alcoholic liver, *etc.*, and especially alcohol consumption. The high activity of GGT in males may be related to alcohol consumption to a certain extent, which shows that its clinical value cannot be ignored, especially for males (*30*). However, the relative deviation of RIs for GGT was smaller than the RCV, and there was no significant difference from the RIs reported by the national standard. Enzyme ALP is closely related to bone growth. The activity was at a relatively stable level in the elderly. The reason may be that the development of bone growth had already reached a stable stage before the onset of old age and therefore showed no large fluctuation in the elderly (*31*). ALP activities were higher in females than that in males. The changes in postmenopausal hormone concentrations can make bone metabolism reach a high transition state, which can increase bone turnover (*32*). This may be the main reason for the higher RIs in females. However, the UL and LL of ALP were different from those studies. It was inferred that this difference may be caused by climate, environment, lifestyle, or diet structure (*15*). In this study, concentrations of TBIL were higher than those in other studies. The proportion of OOR (out of range) values for TBIL was 20.6%. This means 20.6% of enrolled study participants would be regarded as having abnormal results, and therefore more likely to conduct many repeated but unnecessary tests. It can be considered unethical because they put an extra burden on the shoulders of a person whose life should be made as comfortable as possible in this phase of life. Concentrations of DBIL increased with age in males and females in this study, which showed the same results as those in Asmara (*25*).

Unfortunately, this study still has some limitations. We aimed to establish RIs for the elderly population, so some individuals with underlying diseases or subclinical conditions may be included in the study. Data were distributed unevenly among age subgroups, so more data for participants aged 80 or older should be collected. Despite the limitations, the RIs obtained in this study can provide valuable guidelines and facilitate the interpretation of clinical data in elderly individuals.

In summary, the currently used RIs for liver function tests are no longer applicable for the current Chinese elderly population. This study filled a critical gap, as that there had not been a study based on the LIS by an indirect method for defining specific RIs for elderly individuals in Northeast China. Furthermore, the RIs for liver function in the elderly are different from those in the younger population. Significant differences were also found when compared with national standards and RIs in other countries. It is of great significance to establish gender- and age- specific RIs for the elderly.
